# How do Chaperones Bind (Partly) Unfolded Client Proteins?

**DOI:** 10.3389/fmolb.2021.762005

**Published:** 2021-10-25

**Authors:** Iva Sučec, Beate Bersch, Paul Schanda

**Affiliations:** ^1^ CEA, CNRS, Institut de Biologie Structurale (IBS), Univ. Grenoble Alpes, Grenoble, France; ^2^ Institute of Science and Technology Austria, Klosterneuburg, Austria

**Keywords:** conformational ensemble, holdase, entropy, enthalpy, fuzzy complex, chaperone-client complexes, NMR spectroscopy

## Abstract

Molecular chaperones are central to cellular protein homeostasis. Dynamic disorder is a key feature of the complexes of molecular chaperones and their client proteins, and it facilitates the client release towards a folded state or the handover to downstream components. The dynamic nature also implies that a given chaperone can interact with many different client proteins, based on physico-chemical sequence properties rather than on structural complementarity of their (folded) 3D structure. Yet, the balance between this promiscuity and some degree of client specificity is poorly understood. Here, we review recent atomic-level descriptions of chaperones with client proteins, including chaperones in complex with intrinsically disordered proteins, with membrane-protein precursors, or partially folded client proteins. We focus hereby on chaperone-client interactions that are independent of ATP. The picture emerging from these studies highlights the importance of dynamics in these complexes, whereby several interaction types, not only hydrophobic ones, contribute to the complex formation. We discuss these features of chaperone-client complexes and possible factors that may contribute to this balance of promiscuity and specificity.

## 1 Introduction

Molecular chaperones are the essential components to ensure the protein homeostasis of the cell. Their importance is highlighted by their abundance in the cell: the family of 70 kDa heat-shock proteins (Hsp70) on its own, for example, is estimated to correspond to up to 3% of the total protein mass in eukaryotic cells under non-stress conditions Finka and Goloubinoff (2013). There are many types and isoforms of chaperones in each cell, and they generally are organized in cooperating networks Balchin et al. (2016). A central question in understanding chaperone function is how they interact with the polypeptides they bind, i.e., with their “client” proteins. How do chaperones achieve their ability to interact with many different client proteins efficiently while also retaining some kind of specificity? And how do the interactions between chaperones and their clients enable the clients to be refolded, safely transported or even disaggregated from insoluble forms? During the last few years, several complexes of chaperones with their full-length client proteins have been characterized at the atomic level, and have thereby shed light onto the underlying interaction patterns. In this review, we discuss the features of more than ten different chaperone systems, and provide insight into the interactions of these (predominantly folded) chaperones with their (predominantly unfolded) clients, and on how the balance of different types of interactions (hydrophobic, hydrophilic, electrostatic) may lay the basis for achieving some degree of promiscuity and some specificity. We invite the reader who wants to quickly read only about the general common features that emerge from these examples to jump directly to section 6. We also refer to reviews on various aspects of chaperone-client complexes, e.g., those by Kim et al. (2013), Skjærven et al. (2015), Craig and Marszalek (2017), Hiller and Burmann (2018) or Rosenzweig et al. (2019).

## 2 Basic Chaperone Function and Binding Properties

A basic property of a molecular chaperone is its ability to bind to partially or fully disordered client proteins. When bound to chaperones, these latter proteins are generally not in their native functional 3D structure. All proteins in the cell are, at some stage(s) of their life cycle, in such non-native states. This is obviously the case at the very start of a protein’s presence in the cell: when being translated as an unfolded chain on the ribosome, the nascent chains may bind to chaperones before reaching their native fold. Other instances where chaperones are essential are when proteins unfold or misfold. Spontaneous unfolding may arise due to the fact that folded proteins are often only marginally stable, and the disruption of a few interactions within the crowded cellular environment can favor unfolded states [Christiansen et al. (2013), Gershenson et al. (2014)]. Moreover, some inherently insoluble proteins are produced in the aqueous environment of the cytosol, and need to be transported to a different cellular compartment; for example, membrane proteins destined to the bacterial outer membrane or the mitochondrial or chloroplast membranes rely on a suite of chaperones for their transport and insertion into the respective membranes (discussed in section 5.1, section 5.2.1 and section 5.2.2).

The basic ability to interact with disordered proteins is common to all chaperones, at least in some of the conformational states that a given chaperone can adopt. This property is often referred to as “holdase” activity. Some chaperones are assumed to possess primarily, if not exclusively, a holdase activity; this is the case for prefoldin [Vainberg et al. (1998), Arranz et al. (2018)], mitochondrial TIM chaperones [Höhr et al. (2015), Becker et al. (2019)] and bacterial periplasmic chaperones [Skp, SurA and Dsb Goemans et al. (2014), Thoma et al. (2015)]. The role of these holdases is to safeguard their client from aggregation, and hand the protein to other downstream factors, such as other chaperones or insertases or degradation machineries (proteases). The case of J-domain proteins is somewhat particular: they exhibit a holdase function, and relay their clients to Hsp70 chaperones, and they also act upon the Hsp70 chaperone by enhancing the ATP hydrolysis of Hsp70 [Silver and Way (1993), Kampinga and Craig (2010)].

**FIGURE 1:**
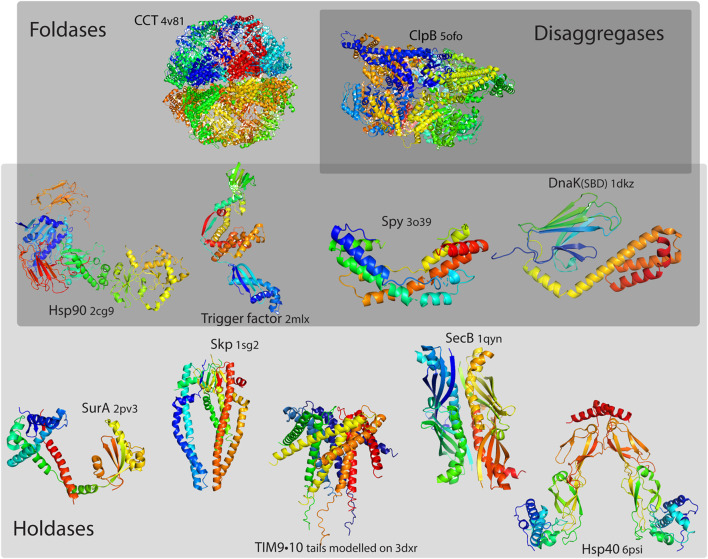
Selected examples of molecular chaperones classified by their chaperoning properties. While all chaperones could be considered as holdases, with the function of binding its structurally unstable client and preventing its aggregation, foldases have an additional function of assisting the client protein folding to its native state, and disaggregases have an additional function in dissociating protein aggregates. Certain chaperones could have multiple functions. Delineating strictly between these functions is hardly possible, as chaperones may have different functions depending on their clients; thus, the frontiers are not to be seen as strict boundaries.

A chaperone that is able to assist the protein folding to its native state, is often described as having an additional “foldase” activity. The notion of foldase comes with the idea that the chaperone plays an active role, exerting some kind of force on its client Nunes et al. (2015). However, the distinction between a holdase and a foldase is not straightforward. Some proteins that are often assigned a foldase function may be rather passive: a client protein may exploit the properties of the chaperone surface to facilitate its refolding (on the chaperone surface), or its unfolding, followed by spontaneous refolding [He et al. (2016), Stull et al. (2016)]. Few selected examples of molecular chaperones classified by their chaperoning properties could be seen in Figure 1.

### 2.1 ATP-Driven Chaperones: Using ATP to Alter the Chaperone While in Action

Some chaperones exploit furthermore an ATPase activity for their function. As in many motor proteins, ATP hydrolysis is exploited to drive conformational transitions in chaperones. A prominent example is Hsp70, in which ATP hydrolysis triggers a conformational transition which leads to a strongly increased client-protein affinity [Zhang and Zuiderweg (2004), Mayer and Bukau (2005)]. The ATP-driven structural transitions that some chaperones undergo can be seen as switches, by which a given chaperone changes its properties and, thus, its ability to act as a holdase. For example, in the large, barrel-shaped group II Hsp60 chaperonins (thermosome, TRiC), client binding occurs in the open state, at hydrophobic binding sites close to the barrel entry; it has been proposed that in the course of the ATP-driven structural transition, these sites become partly buried, which changes the environment of the client and presumably favors its refolding within the (predominantly hydrophilic) chamber, and its subsequent release to the cytosole [Kafri and Horovitz (2003), Meyer et al. (2003), Bigotti and Clarke (2005), Spiess et al. (2006), Reissmann et al. (2007), Nakagawa et al. (2014), Jin et al. (2019)]. Note that this is not the only mechanism by which Hsp60 assists refolding of its clients: refolding outside the chamber is another possible mechanism (Priya et al., 2013). Also other chaperones can employ different mechanism of chaperoning depending on the client and the clients folding pathway (reviewed in Koldewey et al., 2017 on the examples of Hsp60, Hsp70 and Spy).

It is worthwhile noting that it is a common mis-conception that the energy released upon breaking the bond to the γ-phosphate group in ATP is what drives large-scale conformational change. In fact, as any bond breaking, cleaving of γ-phosphate group in ATP does not release energy but rather requires it. This reaction is thermodynamically possible because the phosphoanhydrile bonds are relatively weak and require less energy to break them than the energy released when stronger covalent bonds are formed in the product(s). Breaking the bond of the γ-phosphate group in ATP by nucleophilic attack from water (hydrolysis) or some electron-rich species, most commonly enables the energetically unfavorable reaction to occur by reaction coupling; for example, a phosphorylated product of one reaction is used as a reactant in the second reaction (Berg et al., 2002). In biological systems there are several ways ATP drives the conformational change and subsequently the activity of certain proteins. One of the examples is the sodium-potassium pump that undergoes its first conformational change upon ATP binding, the second conformational change once the protein gets phosphorylated by the γ-phosphate group upon cleavage of ATP and the last one induced by proteins de-phosphorylation. [reviewed in Jorgensen et al. (2003)]. What drives structural transitions of other ATP-fueled machines, such as the ATP-driven chaperones, is the fact that upon hydrolysis two new species, ADP and phosphate, are generated. Their binding properties and charges differ from those of ATP, and these altered properties of the complex drive a conformational rearrangement of the protein [Bagshaw and Trentham (1973), Hwang and Karplus (2019)].

In the present review we focus on ATP-independent chaperones, and their properties as holdases; as stated above, the holdase activity is common to all chaperones, including ATP-fueled ones.

## 3 Chaperone-Client Complexes and the Balance of Stability vs. Ease of Release

The complexes of chaperones with their clients need to fulfill contradicting requirements: on one hand the complexes, at least those of some holdase chaperones, need to be at least somewhat stable, such that the client protein does not spontaneously detach from the chaperone. This property is important particularly for “transfer chaperones”, which accompany highly aggregation-prone clients, such as membrane proteins, to their target insertase or translocase. Spontaneous detachment of the client before the complex reaches its destination may lead to aggregation of the client. On the other hand, release of the client, for example the handover to a downstream insertase or the release of the natively re-folded client, should proceed without a significant energy barrier. From some chaperones (e.g., Spy, see below), the clients detach spontaneously once they reached a conformation allowing the detachment; other chaperone–client complexes dissociate once they reach, e.g., a membrane-protein insertase or translocase, and need to detach without significant energy barrier. (The relay often proceeds without energy input from ATP hydrolysis, e.g., in the bacterial periplasm or the mitochondrial intermembrane space.) Chaperones, therefore, must meet the contradicting requirements of stability and absence of significant energy barriers for dissociation [Hartl et al. (2011), Burmann et al. (2013), Hiller and Burmann (2018)].

As discussed here, using recent examples that have been characterized at the structural level, dynamics within the bound state is the way how this apparent contradiction can be resolved. In this sense, chaperone–client complexes may be seen as “fuzzy” complexes. This term, introduced by Fuxreiter and Tompa Tompa and Fuxreiter (2008), refers to protein-protein complexes in which at least one of the two proteins remains dynamic while bound. In many reported cases the bound client is disordered, and bound to the chaperone as an ensemble of inter-converting states.

Another example of importance of dynamics in chaperone-client interaction is increase in flexibility of the linker loop at the substrate interface of a chaperone Spy, which is proposed to increase promiscuity of Spy Horowitz et al. (2016).

## 4 Specificity vs. Promiscuity

The complexity of living organisms relies on the promiscuity of proteins: enzymes capable of processing a range of substrates or receptors able to recognize different molecules, and also the molecular chaperones, which are able to bind to a range of client proteins. Promiscuity is essential, because if each chaperone was highly specific to a small set of client proteins, the energetic cost of maintaining many different regulatory networks would be very high Cumberworth et al. (2013).

Promiscuity is often assumed to be the rule for chaperones; trigger factor, for example, has a substrate proteome with more than 170 members Martinez-Hackert and Hendrickson (2009); the TIM9.10 chaperone binds at least 40 different proteins; the familiy comprising the 70 kDa heat-shock protein (Hsp70) and Hsp90 family has a very wide clientome that covers at least 20% of the yeast proteome, for example Taipale et al. (2010).

Despite the presence of many chaperones in the cell, each capable to bind a broad range of clients, cellular experiments generally reveal preferences, and not all chaperones bind a given client. This can be nicely illustrated with the example of mitochondrial protein import. Herein, proteins which are destined to the mitochondria but produced in the cytosol need to be guided along the entire way; this is particularly important for mitochondrial membrane proteins, because of their strong tendency to aggregate. A central question in the mitochondrial import field is which chaperones are responsible for the transport of the newly synthesized mitochondrial precursor proteins from the cytosolic ribosomes to the mitochondria.

Insightful studies Jores et al. (2018) have revealed, for example, that newly synthesized outer-membrane β-barrel porins associate with Hsp70 and Hsp90 chaperones as well as with a certain number of Hsp40 chaperones (Ydj1, Sis1, and Djp1) and Hsp104, but not other general chaperones such as Hsp60 or 14-3-3. When doing the same assay with mitochondrial inner-membrane proteins, Hsp70 and Hsp90 are again found to associate, but the levels of associated Hsp40 chaperones differ from the ones found to associate with the β-barrel forming porin. This kind of experiments suggests that Hsp70 and Hsp90 interact with client proteins mainly by hydrophobic interactions, but that the Hsp40 association is based on more subtle differences in their substrate proteins, allowing also to fine-tune the specificity.

Given such kind of experimental findings, the central question is: what are the structural or sequence properties of the client proteins which make one chaperone bind but not another one? And how does the chaperone recognize these differences?

Unfortunately, these questions are not well understood. A few rather rare cases are known where a chaperone is highly specific, with only one Szolajska and Chroboczek (2011) or few clients Kuehn et al. (1993); in these cases, the recognition is achieved by complementary surfaces and a well defined binding site on the client. For example, the periplasmic holdase chaperones PapD and FimC from pathogenic bacteria, are specific for the pilus forming sub-units Sauer et al. (2000) and they interact with their clients via the donor-strand complementation mechanism. Such surface-/strand-complementarity resembles the situation of complexes formed between folded globular proteins. These cases are rather rare and not representative of most chaperones.

For most chaperones, identifying the binding motif, or even clarifying the clientome, is not as easy. In the clients of the Hsp70 chaperone family, a binding motif has been identified. It consists of a hydrophobic core and two flanking regions with basic residues Rüdiger et al. (1997); this motif is indeed very abundant in most proteins. However, for the very important Hsp90 family and its very large clientome, bioinformatic analyses have not been able to identify a specific binding motif Taipale et al. (2010). In some cases, such as the small TIMs discussed in section 5.1, preferences for binding one rather than another client is based on a combination of interaction types within the same binding groove or on different binding sites: a hydrophobic interaction with one binding interface, and a polar/charge-based contact at a separate binding interface.

The specific recognition of clients is related to the process by which clients are targeted to organelles within the cell; for example, precursor proteins destined to chloroplasts or mitochondria need to be recognized by a set of chaperones/receptor domains for their import. In some cases, these targeting signals are very well defined amino acid sequences von Heijne (2002). However, many targeting sequences are less well defined and scattered over the primary structure, that share certain physical properties rather than exact amino acid sequence. For example, mitochondrial preproteins targeted for the matrix carry an amphiphilic helix as a cleavable recognition signal, however the only similarity between the targeting signal of different proteins is that one side of the helix is positively charged whereas the other is hydrophobic Maduke and Roise (1996). Chloroplast outer membrane proteins carry a targeting signal, rather than an exact targeting sequence, recognized by the cytosolic AKR2 (Ankyrin repeat protein) chaperone. In addition to the importance of moderate hydrophobicity of the targeting signal, positively charged residues flanking transmembrane domains are important for specificity, and if these positive charges are missing, the preproteins are targeted to the plasma membrane rather than to the chloroplast Lee et al. (2011).

The mechanisms by which a client is selected by a chaperone and not bound by another one are far from being solved. Deciphering the recognition mechanisms is complicated by the fact that these complexes are often highly dynamic. Therefore, specific contacts with which folded proteins recognize each other, e.g., salt bridges, tend to be short-lived. It is currently poorly understood how specific recognition is compatible with the higly dynamic character of the chaperone–client complexes. The general mechanisms that underlie these complexes, described in the examples below, may nonetheless provide possible routes how specificity may be achieved.

## 5 Lessons Learned From Atomic-Level Studies of Chaperone Complexes

### 5.1 Chaperoning in the Mitochondrial Intermembrane Space

The vast majority of the mitochondrial proteins are imported into the organelle in a post-translational manner as precursor proteins, that are recognized either by a cleavable pre-sequence or by an internal targeting sequence. Cytosolic chaperones transport these precursor proteins to the mitochondrial entry gate, the translocase of the outer membrane (TOM) complex [reviewed in Becker et al. (2019)]. Depending on their final destination, the precursor proteins are then either inserted into the outer membrane from the mitochondrial outside, or directly relayed to the translocase of the inner membrane (TIM23 complex), or transferred across the intermembrane space (IMS) to an insertase in the inner or outer membrane. Here we focus on this latter process and its associated chaperones, namely the translocation of membrane-protein precursors in the intermembrane space. The so-called small TIM chaperones are responsible for this safe translocation of the highly aggregation-prone membrane-protein precursors across the aqueous IMS compartment [Koehler et al. (1998a),Koehler et al. (1998b), Wiedemann et al. (2001), Vergnolle et al. (2005)]. In their apo state, these chaperones form hetero-hexameric complexes of ∼ 65 kDa, composed of alternating subunits of Tim8 and Tim13 [TIM8.13, Beverly et al. (2008)] or Tim9 and Tim10 [TIM9.10, Webb et al. (2006)], or Tim9, Tim10 and Tim12 [TIM9.10.12, Gebert et al. (2008)]. It is interesting to note that the TIM chaperones are in continuous exchange, whereby subunits co-exist between the hexameric state (predominant at ambient temperature) and monomeric forms. At room temperature, approximately 10% of the Tim9 and Tim10 subunits coexist as monomers, together with the hexameric TIM9.10 complex. NMR experiments have established that the integration of subunits into the hexamer (and release of subunits from the hexamer) is slow, on time scales of many tens of minutes Weinhäupl et al. (2021).

**FIGURE 2:**
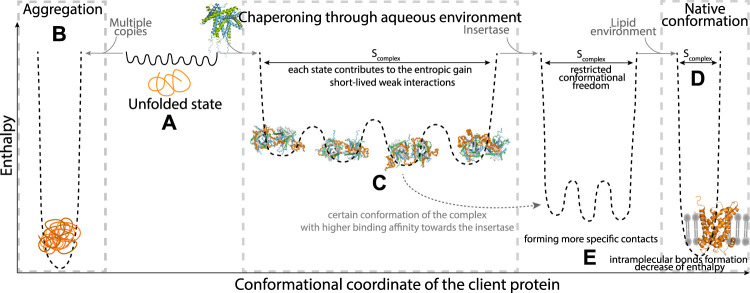
Schematic view of the thermodynamic properties of a client protein exemplified for a membrane-protein client (represented in orange). In the unfolded state of the client **(A)**, the polypeptide exists in an ensemble of multiple rapidly-interconverting conformations. Depending on the concentration of the client (presence of multiple copies) or the presence of a chaperone, the conformational landscape carries different energetic properties. Aggregation, shown on left **(B)** is enthalpically favourable due to multiple intra- and inter-molecular hydrophobic interactions. In the presence of a dedicated chaperone, shown in the middle **(C)**, the chaperone and client form favorable interactions (enthalpic contribution), and as the client generally stays highly dynamic there is no (or little) entropic cost, i.e., the entropy (S_complex_) remains large, comparable to the unfolded state shown in section A of this figure. For the membrane-protein clients, the pathway to the fully folded state **(D)** involves engaging with an insertase, which promotes folding of the client **(E)**. Certain conformations out of the complex ensemble may have higher affinity towards the insertase. The interaction of client and insertase may lead to a step-wise dissociation of the client from the chaperone and formation of more specific contacts with the insertase. The lower conformational entropy (S_complex_) may be compensated by favorable enthalpic interaction, or entropy gain from release of structured water molecules.

As the only known chaperone system of the mitochondrial IMS, TIM chaperones are crucial for the recognition and transfer of most of the mitochondrial membrane precursor proteins Morgenstern et al. (2017). They recognize a broad range of membrane proteins and transfer them in an unfolded state from the mitochondrial outer membrane pore (TOM, with its central pore formed by Tom40), through the aqueous mitochondrial intermembrane space, towards the insertases of the inner membrane (TIM22) or outer membrane (SAM) [Koehler et al. (1999), Bauer et al. (2000), Rehling et al. (2003), Paschen et al. (2003), Kozjak et al. (2003), Gentle et al. (2004), Lithgow and Schneider (2010)]. As for many chaperone-substrate pairs, where the substrate is often aggregation prone, protecting the clients from misfolding and aggregation requires that these complexes do not dissociate spontaneously, i.e., the overall affinity of chaperone and client needs to be relatively strong. It is difficult to experimentally determine dissociation constants (K_d_) because one cannot obtain these complexes by simply titrating solutions of chaperone and client due to the insolubility of the membrane-protein precursors. For the small TIM chaperones, no experimental K_d_ values are available. However, it has been experimentally shown that the transfer of client proteins (membrane proteins of the inner membrane, so-called mitochondrial carriers) from one TIM chaperone to another takes approximately 4 h Weinhäupl et al. (2018), indicating high stability of the client-TIM complex in the absence of the downstream insertase of the inner membrane. In the cell, release at the insertase is presumably energetically more favorable (Figure 2), as the time scale for import into mitochondria is rather in the minutes time scale Wiedemann et al. (2006). It is likely that interactions of a part of the (highly dynamic) precursor protein with the insertase complex lowers the energy barrier for release (Figure 2).

When TIM chaperones bind clients, they do not undergo significant changes of their structure, nor of their backbone or sidechain dynamics, as revealed by NMR methods Weinhäupl et al. (2018). Interestingly, also the above-mentioned exchange of subunits between monomers still exists when the client is bound to the hexameric TIM9.10. Subunits still enter and exit the hexamers, with a time scale similar to the apo chaperone Weinhäupl et al. (2021). The very different size and thus NMR properties of the chaperone-client complexes and monomers makes it difficult to quantify the populations with precision, but the data suggest that even the ratio (monomeric subunits vs. hexamer) is similar to the apo state. This is different to the Skp chaperone or DegP (see below), which assemble upon client binding.

**FIGURE 3:**
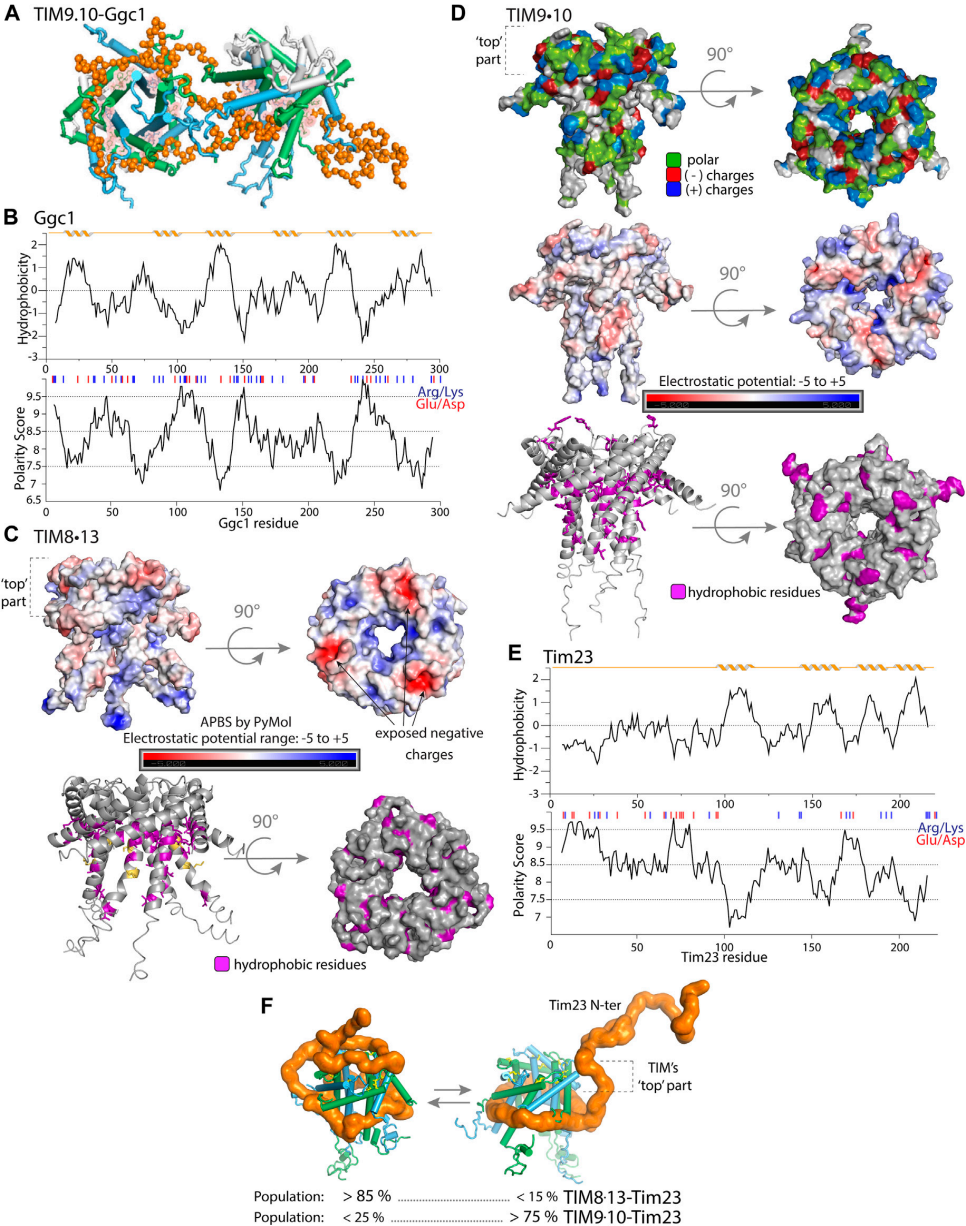
Properties of mitochondrial intermembrane space chaperones and their selected clients. **(A)** Representation of the structure of TIM9.10 (blue/green) holding full-length client GDP/GTP carrier (Ggc1, orange), as reported in Weinhäupl et al. (2018). In contrast to the complexes of TIM8.13, the entire client protein is bound to two chaperone molecules in the hydrophobic binding cleft of the chaperone. Figure adapted from Sučec et al. (2020). **(B)** Properties of full length Ggc1 client protein. Hydrophobicity and polarity predictions were calculated as mentioned for Tim23. **(C)** Properties of TIM8.13 chaperone (PDB 3cjh). In the upper panel the electrostatic potential is mapped on the TIM8.13 structure (model with extended tentacles), calculated with PyMOL APBS plugin. The molecule was prepared with the pdb2pqr method and the script was run with the standard setup (range of ±5.00, grid spacing 0.5). The top part of the chaperone shows more exposed negatively charged residues, lower electrostatic potential, compared to the rest of the protein and compared to TIM9.10 (panel E). Mapped conserved hydrophobic residues on TIM8.13 model (purple, **lower panel).** Yellow: polar residues in the conserved hydrophobic positions leading to weaker binding of all hydrophobic clients. **(D)** Properties of TIM9.10 chaperone (PDB: 3dxr). Upper panel: positive (blue), negative (red) charges and polar (green) residues. Electrostatic potential mapped on TIM9.10 model with extended tentacles **(middle panel)**. Lower panel: conserved hydrophobic residues (in the binding cleft and on the “top”) on TIM9.10 model, shown in purple. In the top view, all hydrophobic residues of TIM9.10 are shown. **(E)** Properties of full length Tim23 client protein. Upper plot: hydrophobicity prediction (Kyte-Doolittle scale); lower plot: polarity prediction (Grantham scale, Expasy Bioinformatics Resource Portal). Red and blue bars: negatively and positively charged residues. NMR spectra of the soluble Tim23 N-terminal fragment (residues 1–98) in isolation show the hallmark features of a highly flexible intrinsically disordered protein Sučec et al. (2020). **(F)** Representative conformations of TIM chaperones bound to Tim23 client protein in which the hydrophilic N-tail of Tim23 is either bound or unbound on the ‘top’ part of the chaperone. The best-fit populations of the two classes of states are shown for either TIM9.10 or TIM8.13 as derived from SAXS/MD. Figure adapted from Sučec et al. (2020).

The α-helical inner-membrane protein clients are wrapped around the TIM chaperones, using a cleft that is formed by highly conserved hydrophobic residues. The fact that these hydrophobic patches are in a cleft presumably helps to shield them, such that TIM chaperones do not aggregate by intermolecular hydrophobic interactions. Depending on the client length, a single client can recruit up to two TIM9.10 chaperones for the transfer (Figure 3A), or possibly even more than two chaperones (although this has not been shown yet). The clients are, thus, in extended conformation, which is quite different from the compact “fluid-globule” state that OMPs adopt in the bacterial membrane-protein chaperone Skp (see below). Interestingly, the clients, both α-helical and β-barrel-forming proteins, have some degree of secondary structure; some residual α-helical propensity was detected for inner-membrane proteins Weinhäupl et al. (2018), and for β-barrel clients it was shown that only clients with pre-formed β-turns bind efficiently Jores et al. (2016). For the latter case, this appears intuitive, because in a β-turn of β-barrel outer-membrane proteins one face is hydrophilic and the other is hydrophobic. A β-hairpin conformation would ensure exposure of an entire hydrophobic patch for efficient binding to the hydrophobic cleft of the chaperone.

The bound clients are highly dynamic, adopting multiple inter-converting, short-lived conformations, while staying bound on the chaperone surface, in an unfolded, extended state Weinhäupl et al. (2018). These inter-conversion dynamics occur on a time scale of ca. 1 ms: specific NMR methods probing this time window (relaxation-dispersion NMR) have highlighted extensive conformational exchange in the complex Weinhäupl et al. (2018). The overall high affinity is thus achieved by multiple contributions from weak interactions, primarily within the conserved hydrophobic cleft. The authors have proposed that the dynamics of the client protein in the bound state enable the successful transfer of the client protein to the insertase without significant energy barrier: in some of these inter-converting client–chaperone conformations certain parts of the client protein, those with a higher affinity for the insertase, are transiently detached from the complex. Upon interaction with the insertase the clients are gradually released from the chaperone without significant energetic barrier. The favorable enthalpical contribution of the client’s folding makes this process energetically favorable (Figure 2). The dynamics of these chaperone-client complexes are the key to reconcile two apparently contradicting requirements: high overall stability and a low energy barrier for release. Here, the avidity of many individually weak interactions ensures high overall complex stability, whereas transfer to the downstream insertase proceeds step-wise, breaking only a few, weak interactions at a time, and therefore without the need for a large activation energy to be overcome. This kind of mechanism has been proposed for the functionally similar but structurally very different Skp complexes Burmann et al. (2013).

It has also been investigated which parts of the client interact with and are important for binding to the chaperones. Regarding the transmembrane part of the clients, e.g., the ADP/ATP carrier (Aac) or the GDP/GTP carrier of the inner membrane, the above-mentioned integrated NMR study has provided only limited information on a residue-by-residue basis. The millisecond dynamics of the client within the binding cleft leads to severe line broadening and relatively poorly resolved spectra of the clients Weinhäupl et al. (2018), and has hampered the identification of specific residues of the clients that bind to the cleft. Some information about the relative importance of different parts of the client Aac comes from interaction studies with peptide fragments Curran et al. (2002). Briefly, a peptide scan with 13-residue-long fragments along the sequence of Aac, each overlapping with its predecessor by 10 residues, was performed; the peptide fragments were covalently linked to a cellulose membrane, incubated with TIM9.10, and the quantity of bound chaperone was assessed by immunodetection (α-Tim10). By far the highest complex yield was achieved for fragments derived from the transmembrane helices, while only very small amounts were detected for fragments from the hydrophilic loop regions. Although this data does not provide residue-specific information, it establishes that hydrophobic fragments are important for binding. This finding is expected, given the hydrophobic character of the binding groove on the TIM chaperone.

Analysis of the structural properties of the TIM–client complexes have shed light onto the types of interactions that are crucial for complex formation. The two TIM chaperones, TIM8.13 and TIM9.10, are structurally very similar, and they both have highly conserved hydrophobic residues located in the cleft formed between the inner (N-) and outer (C-terminal) *α*-helices. However, they have different specificity towards the mitochondrial precursor proteins [Weinhäupl et al. (2018), Sučec et al. (2020)]. For membrane-protein clients consisting essentially of transmembrane spanning parts, such as mitochondrial solute carriers of the inner membrane like the ATP/ADP carrier (Aac) Curran et al. (2002) and the outer membrane *β*-barrel proteins [Hoppins and Nargang (2004), Habib et al. (2005)], TIM9.10 has higher binding affinity than TIM8.13 [ca. 10-fold higher Weinhäupl et al. (2018), Sučec et al. (2020)]. However, TIM8.13 has higher affinity for binding membrane-protein clients with an additional soluble and more hydrophilic domain, such as Tim23, the translocase of the inner-membrane (TIM23) complex Davis et al. (2007), and the aspartate-glutamate carriers Roesch et al. (2004). TIM9.10’s native clients are all-transmembrane mitochondrial precursor proteins which are highly hydrophobic, such as the mitochondrial carriers, of which one representative is shown in Figure 3B., although TIM9.10 is also able to bind to e.g., Tim23.

How can this somewhat different client specificity of the two overall very similar chaperones be explained? Two regions on TIM chaperones have distinct properties, and are, thus, presumably responsible for the client specificity. Firstly, certain residues within the conserved hydrophobic cleft are less hydrophobic (Lys, Ser) in TIM8.13 compared to the corresponding positions in TIM9.10 (see the orange residues in Figure 3C, lower panel). As a consequence, TIM8.13 might be less able to hold the transmembrane, hydrophobic parts of mitochondrial preproteins than TIM9.10. In a recent study it was shown that a TIM8.13 mutant, in which these more hydrophilic residues were changed to hydrophobic ones, was much more capable of holding all-transmembrane (TIM9.10) clients Sučec et al. (2020).

A second difference is found in the top part of the chaperones, where TIM9.10 differs from TIM8.13 in polarity and charge (Figures 3C,D). TIM8.13 uses additional hydrophilic interactions for binding the N-terminal region of Tim23 via the top part of the chaperone (Figure 3C). It is noteworthy, however, that single-point mutations in this top part of the chaperones did not swap the client affinities of TIM9.10 and TIM8.13, unlike in the above-described case where the hydrophobic cleft of TIM8.13 was rendered more hydrophobic. This suggests that the interaction with the hydrophilic part of Tim23 involves a more complex pattern than could be resolved by the few mutations introduced Sučec et al. (2020).

NMR data of the soluble Tim23 fragment show that TIM9.10 hardly interacts with this predominantly polar fragment; in fact the detected interaction involves only a patch of hydrophobic residues at the N-terminus of Tim23 (MSWLFG) and a further stretch with increased hydrophobicity Sučec et al. (2020). TIM8.13 interacts much more strongly with the soluble fragment of Tim23, and the interaction involves a stretch of at least 35–40 residues of Tim23.

Taken together, the current view is that the hydrophobic binding cleft of small TIMs enables (promiscuous) binding to the hydrophobic transmembrane parts of the clients, whereby TIM8.13 is less hydrophobic and thus less performing in this binding; additional hydrophilic interactions compensate to some degree for this lower affinity, depending on the client. In the case of Tim23 (Figure 3E), the resulting ensembles of states of the TIM9.10 or TIM8.13 complexes have different population levels, as revealed by SAXS/MD data. Figure 3F recapitulates this situation for Tim23-binding to the two chaperones. The states in which the hydrophilic tail of the client interacts with the hydrophilic top part of the chaperone are much more populated in the case of TIM8.13 than TIM9.10, and these hydrophilic interactions compensate for the inherently lower ability of TIM8.13 to interact via its hydrophobic cleft with the transmembrane part.

### 5.2 Chaperoning in the Bacterial Periplasm

In Gram-negative bacteria, outer membrane proteins (OMPs) are synthesized on the cytoplasmic ribosome and translocated in an unfolded form across the inner membrane by the Sec machinery [reviewed in Komarudin and Driessen (2019), Oswald et al. (2021)]. To reach their final destination, they have to cross the periplasm, an aqueous compartment. At the exit of the Sec machinery, OMPs are taken in charge by periplasm-specific chaperones. The bacterial periplasm is a special and somewhat demanding environment for chaperones, because 1) it lacks ATP, 2) it is an oxidizing environment and 3) it is separated from the outside only by a porous membrane, and is therefore particularly susceptible to changes in the outside. The periplasm of gram-negative bacteria contains ATP-independent chaperones that contribute to the biogenesis of OMPs Sklar et al. (2007): the holdases SurA, Skp, FkpA and PpiD as well as DegP, which has chaperone and protease functions [Arié et al. (2001), Matern et al. (2010), Merdanovic et al. (2011), Ge et al. (2014)]. The membrane anchored chaperone PpiD contributes to the efficient detachment of newly secreted OMPs from the Sec machinery Fürst et al. (2018). Other periplasmic chaperones such as SurA, DegP, and Skp are likely to take over newly translocated proteins from PpiD on their way into the periplasm or to the outer membrane. Outer membrane protein biogenesis in Gram-negative bacteria is reviewed in detail, e.g., in Rollauer et al. (2015), Schiffrin et al. (2017), Tomasek and Kahne (2021).

**FIGURE 4:**
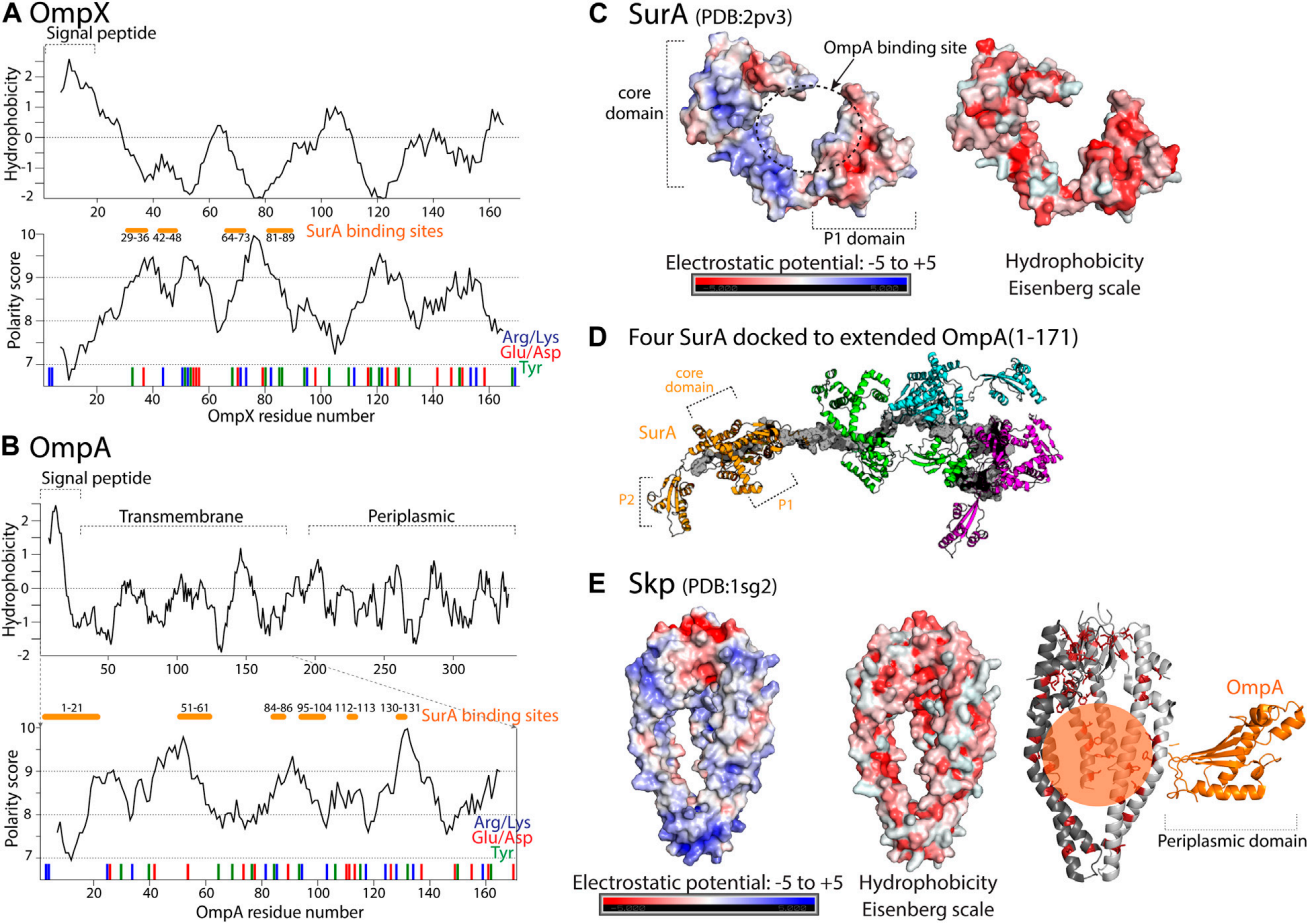
Properties of periplasmic chaperones SurA and Skp and their selected client proteins. **(A)** Hydrophobicity prediction of the full length OmpX client protein based on Kyte-Doolittle hydrophobicity scale is shown in the upper plot. Polarity prediction based on Grantham scale, performed with Expasy, is shown in the lower plot as a function of OmpX residue number. In red and blue bars, position of negatively and positively charged residues is shown. Position of tyrosine residues is shown with green bars and proposed binding site to SurA Marx et al. (2020b) is shown with orange bars. **(B)** Upper plot shows hydrophobicity prediction of the full-length OmpA client protein. In the lower plot polarity prediction for residues 1–171 of OmpA is shown based on Grantham scale. In red and blue bars, position of negatively and positively charged residues is shown. Position of tyrosine residues is shown with green bars, as they are proposed to be involved in the binding to SurA Marx et al. (2020b). **(C)** Electrostatic potential and hydrophobicity (Eisenberg scale) plotted onto the core and P1 domains of SurA [in the open/bound conformation Xu et al. (2007)]. **(D)** Model of the SurA-OmpA (1–171) complex with four full-length SurA (colored molecules) docked to expanded OmpA (shown in gray). Figure adapted from Marx et al. (2020b). **(E)** Electrostatic potential and hydrophobicity (Eisenberg scale) plotted on the surface of the Skp chaperone. On the right, representation of OmpA-Skp complex whith the client protein shown in orange. The periplasmic domain of OmpA is proposed to be folded and not bound to Skp while the β-barel domain remains unfolded in the Skp binding cleft Walton et al. (2009). The whole transmembrane part of OmpA is bound within the Skp tentacles through multiple electrostatic and hydrophobic interactions Qu et al. (2009). In the complex, hydrophobic residues of Skp are shown as sticks colored in red.

The bacterial periplasmic chaperones Skp (Seventeen Kilodalton Protein; Chen and Henning (1996)) and SurA [Survival factor A, Behrens et al. (2001), Bitto and McKay (2002)] share the pool of OMP clients; initially, they appeared to have redundant function in escorting the OMPs to the BAM complex of the outer membrane [Sklar et al. (2007), McMorran et al. (2015)]. Recent work Wang et al. (2021) indicates that the SurA chaperone, with the PPIase (peptidyl-prolyl *cis*-trans isomerase) activity Sklar et al. (2007), has a role in targeting OMPs to the BAM complex, while the chaperone Skp delivers unintegrated OMPs to the DegP for their degradation. The client proteins that have been extensively used as models for interactions, OmpX and OmpA, share similar hydrophobic and polar properties of their amino-acid sequence in the transmembrane parts (Figures 4A,B); OmpA has an additional soluble periplasmic domain on the C-terminus (but several studies used only the TM part as client).

#### 5.2.1 SurA

SurA is the key chaperone for insertion of OMPs into the OM by the help of the BAM complex Sklar et al. (2007). It is composed of three domains: the core domain which is formed by the N- and C-terminal regions and two central PPIase domains (P1 and P2). The crystal structure of SurA has been determined in its apo form Bitto and McKay (2002). In this structure, P1 is bound to the core domain whereas P2 is connected to the core domain by an extended linker. In solution, SurA appears to be monomeric [Calabrese et al. (2020), Marx et al. (2020a)] and samples at least two different conformational ensembles that are distinct from the crystal structure Calabrese et al. (2020). The different conformations are in dynamic interconversion on a submillisecond time scale. The hydrodynamic properties of SurA reveal a radius of gyration compatible with one of the two PPIase domains being spatially separated from the core structure. SurA switches between a major P1-closed and a minor P2-closed state [Marx et al. (2020a), Jia et al. (2020)]. It has been shown that the two PPIase domains compete in a non-allosteric manner with each other for binding to the core domain Marx et al. (2020a).

The presence of either P1, P2, or both PPIase domains was shown not to be required for chaperoning activity of SurA, as all three mutants fully complement Δ*surA* OMP assembly phenotypes in an otherwise unmodified genetic background Soltes et al. (2016). Whereas other chaperones (Skp, DegP) assemble to form a cage-like cavity for client binding (see below), SurA does not oligomerize. Client binding and the client complexes have been studied using different techniques including NMR Thoma et al. (2015), single molecule flourescence Chamachi et al. (2021) and cross-linking mass spectrometry [Calabrese et al. (2020), Marx et al. (2020b)] experiments. It has been shown that SurA recognizes substrates with a preferential Ar-X-Ar motif (Ar: aromatic amino acid, X: any type of residue) Bitto and McKay (2003). Such a tripeptide is found at the C-terminus of many OMPs and this so-called *β* signal has been proposed to play a role in the recognition of OMP clients by the BAM complex [Hennecke et al. (2005), Wang et al. (2021)].

NMR and single-molecule fluorescence have shown that the SurA-bound OMP client proteins form a rapidly exchanging conformational ensemble with exchange rate constants on the microsecond timescale [Thoma et al. (2015), Chamachi et al. (2021)]. Crosslinking mass spectrometry experiments [Calabrese et al. (2020), Marx et al. (2020b)] revealed that SurA binds the OMPs in a groove formed between the core and P1 domains. This groove forms when the two PPIase domains are simultaneously dissociated from the core Marx et al. (2020b) and the role of the P2 domain is regulating the interaction between the P1 and the core domains Calabrese et al. (2020). The OMP binding site on SurA is large enough to accommodate an entire transmembrane *β*-strand or *β*-hairpin. The bottom of the groove is formed by a 30 Å-wide hydrophobic stretch and is positively charged (Figure 4C, left panel). Regions of the core and the P1 domain outside of the groove bear negative charges, suggesting that electrostatic interactions contribute to the complex formation between SurA and its clients. These features may be required to accommodate the alternating hydrophobic–hydrophilic patterns of extended OMP transmembrane domains. Interestingly, the non-client protein OmpLA showed much less cross-linking products than the SurA clients OmpA (transmembrane domain) and OmpX, suggesting that SurA shows substrate specificity in solution, independently of co-chaperones Marx et al. (2020b). SurA-bound OMPs are in an extended conformation [Marx et al. (2020b), Chamachi et al. (2021)]. Several binding regions of SurA, on both OmpX and transmembrane domain of OmpA, were detected (Figures 4A,B, shown as orange bars); the importance of multiple conserved Tyr residues in the binding site has been suggested Marx et al. (2020b). It is noteworthy that the preferred contact sites do not occur at the site of the *β*-signal. This suggests that this fragment, important for the recognition by the BAM complex is free, flexible and thus available for protein-protein interactions.

SurA binds the unfolded OMPs with a dissociation constant (K_d_) of a few hundred nanomolar [Bitto and McKay (2003), Bitto and McKay (2004), Wu et al. (2011), Chamachi et al. (2021)]. This overall strong binding of SurA and the client is achieved through many weak and not very site-specific interactions He et al. (2020). A recently proposed model Marx et al. (2020b) is that multiple SurA can bind to the transmembrane domain of OmpA, keeping it in an extended, insertion-competent state (Figure 4D). Such higher order complex formation may depend on the length of the client protein, as also observed for the small TIM chaperones [Figure 3A; Weinhäupl et al. (2018)]. FhuA, which forms a 22 strand *β* barrel showed a close to 1:2 stoichiometry in complex with SurA Thoma et al. (2015). Effective transfer of the client to the BAM complex requires the BAM interacting region of the OMP to be free and flexible [Wang et al. (2016), Marx et al. (2020b), Wang et al. (2021)]. Since the client samples multiple conformations during its interaction with the chaperone He et al. (2020), it can be expected that some of those client conformations have higher binding affinity towards the BAM complex and the dissociation from SurA and insertion into the membrane can continue in a similar way as seen for TIM chaperones.

It has been proposed that SurA may not hold the OMP client continuously, but rather bind and unbind rapidly and repeatedly. Marx et al. (2020a) proposed that the binding to the OMP may be faster than the collapse of the OMP to a molten-globule state, as the OMPs are not very hydrophobic. This “kinetic trapping” model awaits further experimental investigation.

#### 5.2.2 Skp

The 17 kDa protein (Skp) chaperone represents a pathway for OMP transport across the bacterial periplasm that is parallel to SurA. The crystal structure of Skp shows a trimeric oligomeric state with a “jellyfish”-like architecture [Korndörfer et al. (2004), Walton and Sousa (2004)]. Within the trimer, a nine-stranded *β*-barrel is formed to which each monomer contributes three *β*-strands (the trimerization interface) and a long, *α*-helical “tentacle,” made of two *α*-helices in a coiled-coil arrangement. These helices are highly dynamic in solution and sample a large conformational space Holdbrook et al. (2017), thereby allowing a drastic increase of the cavity in an ATP-independent manner. Interestingly, a recent study proposes an Skp activation mechanism that involves a monomer to trimer transition induced by the unfolded client protein. wtSkp has been shown to exist in a monomer-trimer equilibrium [Mas et al. (2020), Sandlin et al. (2015)], the monomer being intrinsically disordered Mas et al. (2020). Fully monomeric Skp mutant proteins were unable to bind unfolded client proteins, whereas NMR Mas et al. (2020) and smFRET Pan et al. (2020) studies showed substrate-induced trimer formation for Skp. It seems that simultaneous contacts of the unfolded client protein with all three Skp subunits stabilizes the trimer by avidity. This coupled folding and oligomerization mechanism may ensure the tight regulation of Skp activity in the periplasm Mas et al. (2020).

Skp-OMP complexes have been extensively studied by NMR, and it represented the first such atom-specific study of a full-length client bound to a chaperone Burmann et al. (2013). NMR spectra acquired on trimeric Skp show only a single set of NMR resonances for the three subunits, demonstrating that on time average each subunit samples the same conformational space. Binding of client protein to the trimer induces a transition from a very flexible state Holdbrook et al. (2017) to a more rigid state of the long *α*-helical tentacles Burmann et al. (2013). The decrease in dynamics within the helices forming the substrate-binding cavity is thought to keep the uOMP within the cavity. The three-fold symmetry of the Skp subunits remains intact in the Skp-OMP complexes, which shows that the complex must be in a dynamic equilibrium of multiple states on a sub-millisecond time scale; non-dynamic binding would break the symmetry. The interaction strength of Skp with client proteins has been determined experimentally, and lies in the nanomolar K_D_ range [Qu et al. (2007), Wu et al. (2011), Chamachi et al. (2021)]. Skp-client protein complexes were shown to have global lifetimes of several hours *in vitro* Burmann et al. (2013). Although it may at first sight appear counter-intuitive to have such a high affinity in such a dynamic complex, it is the avidity of the multiple interactions, each individually weak and thus short-lived, which allows for the high overall affinity.

NMR data, including paramagnetic NMR that probe distances from a paramagnetic tag, suggest that, despite its high dynamics, the client protein adopts a more compact state within the Skp cavity than expected for a urea-denatured protein. From such NMR data, an average radius of 21 Å for OmpX in the Skp cavity has been found, whereas in 8 M urea the corresponding radius is more than two-fold larger Burmann et al. (2013). The authors proposed the term “fluid globule” to describe this very compact, yet highly dynamic nature of the client.

Recently, smFRET studies have revealed that Skp-bound uOMP had a lower energy transfer efficiency than aqueous OmpX Chamachi et al. (2021) or OmpC Pan et al. (2020), suggesting chaperone-induced extension of the aqueous, unfolded (but not denatured) OMPs. Pan et al. have estimated the radius of the OmpC-Skp complex to 39 Å Pan et al. (2020) which, assuming a radius of 6 Å for the Skp helices surrounding the binding cavity, seems compatible with the radius of the (shorter) OmpX client of 21 Å, deduced from NMR data Burmann et al. (2013). Skp therefore binds its client protein in a highly dynamic state, which is more compact than the urea denatured protein but more extended than the collapsed, aqueous OMP at pM concentration. Using single molecule FRET spectroscopy, Chamachi et al. found intra-chain dynamics on the µs time-scale for Skp-bound OmpX Chamachi et al. (2021). As stated above, fast inter-converting client conformations (fuzziness of the client) are providing multiple protein-protein interaction sites. These are required for the formation of the holdase-competent Skp trimer and may also allow the ATP-independent transfer to downstream factors, such as BamA or the degradase DegP.

There is no specific motif in the transmembrane domain of OMPs that is known to be the recognition site of Skp. Instead, it is thought that the entire unfolded transmembrane domain is engaged in the interaction. By adapting the size of its binding cage or by recruiting more homotrimer chaperone complexes [Korndörfer et al. (2004), Schiffrin et al. (2016)] Skp is able to bind a broad range of clients of different sizes, including OMPs and periplasmic proteins Jarchow et al. (2008). This ability of clients with different lengths to recruit more or less chaperones is reminiscent of the case of the mitochondrial TIM chaperones or SurA (see above). Positively charged Skp tentacles (Figure 4E) bind the entire transmembrane domain of the OmpA client (Figure 4B) through multiple electrostatic and also hydrophobic interactions, encapsulating it completely, while the periplasmic domain of OmpA is soluble, outside of the Skp binding cavity, and according to its NMR signature in a folded state that resembles the final native state of this domain [Qu et al. (2009), Walton et al. (2009)]. This ability of leaving soluble parts of the client outside the binding cleft/cavity was also found in the case of the mitochondrial TIM8.13/TIM9.10 interacting with the Tim23 client (see section on mitochondrial TIM chaperones, Figure 3F).

Interestingly, different OMPs show very similar behavior in Skp Burmann et al. (2013); furthermore, a given OMP shows a very similar random-coil behavior in different chaperones [Skp and SurA; Thoma et al. (2015)].

#### 5.2.3 DegP

The stress-induced DegP belongs to the High Temperature Requirement A (HtrA) protein family in the bacterial periplasm, where it is important for quality control of outer-membrane proteins. It has an established function in the degradation of proteins via its serine protease activity; it has also been shown to exhibit chaperone properties [Spiess et al. (1999), Clausen et al. (2002), Jiang et al. (2008), Subrini and Betton (2009), Clausen et al. (2011), Sawa et al. (2011)]. The protease function may be the more important one. Binding of unfolded OMPs to SurA or Skp is at the rate 1000-fold higher than binding to the DegP. It has been proposed that this difference in kinetics favors OMP binding to the former two chaperones, thus preventing degradation Wu et al. (2011).

DegP is found to exist in an inactive hexameric form (presumed to be a resting state) in which the so-called LA loop of the PDZ1 domain interacts with the active site L1 and L2 loops from a neighbouring subunit, thereby blocking the access to the catalytic side Krojer et al. (2002). The inactive hexameric form of DegP is converted into 12- and 24-mers upon interaction with client proteins [Krojer et al. (2008b), Jiang et al. (2008)]. This transition requires binding of unfolded substrate protein, where the C-terminus binds to the PDZ1 domain while the cleavage site is presented to the protease domain Krojer et al. (2008a). It has been shown that simultaneous binding of covalently linked PDZ1-binding and cleavage-site degrons is required for efficient formation of the active, dodecameric protease complex Kim et al. (2011). The crystal- and cryo-EM structures of the 24-mer of DegP have been obtained from the proteolytically inactive DegP_S210A_ mutant that lacks the catalytical Ser; the protease-inactive 12- and 24-mer DegP variants were purified in presence of substrate protein Krojer et al. (2008b). The crystal structure of the 1.13 MDa large 24-mer of DegP Krojer et al. (2008b) shows the formation of a large cavity interior of the 24-mer forming wide pores (up to 35Å) and 24 proteolytic sites that could be accessed only by the encapsulated client protein. It is interesting to note that oligomerization, in this case to 12- or 24-mers, plays an important role for activating the chaperone; dynamic oligomerization has been found already in Skp and TIM chaperones (see above).

In the context of this review, it is particularly interesting to consider the state of the encapsulated protein and the way it may interact with the chaperone. For the DegP case, the information available about the state of the encapsulated client protein and the binding motif(s) is somewhat indirect, as there are no NMR or smFRET studies available to date. Intriguingly, Krojer et al. (2008b) have proposed that the OMPs may be present in a folded state inside the cavity. This proposition comes from several observations. The co-purified substrates with wild-type DegP were stable over tens of minutes; as unfolded model proteins are digested over this time scale, their interpretation is that the OMPs may be folded. Furthermore, a characteristic shift in SDS-PAGE mobility, often used as a signature of folded OMPs, suggested that at least 50*%* of the encapsulated OMP clients had some residual tertiary structure. In the same study, cryo-EM reconstitution of co-purified OMP and protease-inactive DegP 12-mer variant showed an electron density in the center of the cavity, which was interpreted as belonging to a folded OMP encapsulated. However, due to low resolution of the electron density map (28 Å), it is difficult to make definite statements on the folding state, or to characterize the interactions formed between chaperone and client.

#### 5.2.4 DegQ

A study of the related HtrA protein DegQ, another degradase of the bacterial periplasm with dual protease and chaperone functions, provides interesting further insight into this family. Like DegP, DegQ exists in a resting hexamer state and can form 12- and 24-mers upon substrate interaction. DegQ does not play a role in OMP biogenesis but targets rather soluble proteins. Malet et al. used chemically unfolded and reduced lysozyme, or a short peptide that binds to the PDZ1 domain, to trigger the oligomerization to the 12-mer or 24-mer state Malet et al. (2012). The cryo-EM structure (at ca. 12–14 Å resolution) and mass-spectrometry analysis of a protease-deficient DegQ mutant in its 12-mer state shows that it harbors simultaneously five to six lysozyme molecules. At this resolution it remains difficult to make definite statements about the state of the client or its interaction mode with the chaperone; nonetheless, the identification of rather well-defined lobes is compatible with a lysozyme molecule that is close to its native conformation Malet et al. (2012). Six lysozyme molecules could be fitted into the electron density without clashes. Tryptophan fluorescence measurements suggested a folded state, although a slight shift relative to the Trp spectrum of isolated folded lysozyme suggested that the fold may be altered.

By comparison of the lysozyme-filled DegQ with DegQ that was triggered to form the 12-mer with the short peptide, the authors could localize the lysozyme molecules, and thus infer information about the interacting parts of the chaperone Malet et al. (2012). Several loops have been found in the vicinity of the density that was ascribed to lysozyme [see Figure 1D of ref. Malet et al. (2012)]; these regions (a helix corresponding to residues 251–257, and loop residues 408–413, 31–33 and 58–62) contain predominantly methyl-bearing residues and only few charges (a single Lys is oriented towards the lysozyme).

To summarize, both DegP and DegQ seem to support folded clients within their cavities, although the low resolution of the available data hampers precise statements about the interaction modes.

#### 5.2.5 Spy

The ATP-independent periplasmic chaperone Spy (spheroplast protein y) can bind to both native (folded) and non-native proteins, but with higher affinity for the latter. Ability to bind both unfolded and folded clients enables client folding while bound to the chaperone Mitra et al. (2021). It can be imagined that this ability could disrupt normal cell function by interacting with folded proteins. However, in the cell, levels of Spy are well controlled and Spy is only up-regulated in the stress conditions induced by protein aggregation Quan et al. (2011).

This stress-induced dimeric chaperone forms a cradle-like structure through an anti-parallel coiled-coil interaction of two 16 kDa monomers [Quan et al. (2011), PDB ID: 3o39]. Spy binds unfolded periplasmic or outer-membrane proteins with its cradle-shaped binding site, formed mostly by positively charged residues, and allows for their full folding while they are bound Stull et al. (2016). The structural and dynamical properties of Spy with a client, the small helical protein Im7, have been investigated independently by several groups, using NMR spectroscopy, MD simulations, crystallography and other biophysical methods [Salmon et al. (2016), Horowitz et al. (2016), He et al. (2016)]. Im7 is an interesting case because in the absence of its cognate binding partner (colicin E7), several residues at its binding interface are in energetic conflicts, i.e., they are restrained such that they cannot engage in the energetically favorable interactions with other residues, a situation termed local frustration Ferreiro et al. (2014). The Spy-Im7 complex is an instructive example for understanding chaperone-client interactions. It is also an interesting case that highlights the difficulty of studying such complexes by crystallography: the high flexibility of the client and accordingly low electron density renders interpretation of the data difficult, possibly leading to mis-interpretation Wang (2018), which might have challenged one such study Horowitz et al. (2016). This controversy regarding Spy-Im7 crystal structures has been addressed (Rocchio et al., 2019), where the low occupied conformational ensembles of Im7 were reconstituted using selective anomalous labeling and residual electron and anomalous density (READ) method.

**FIGURE 5:**
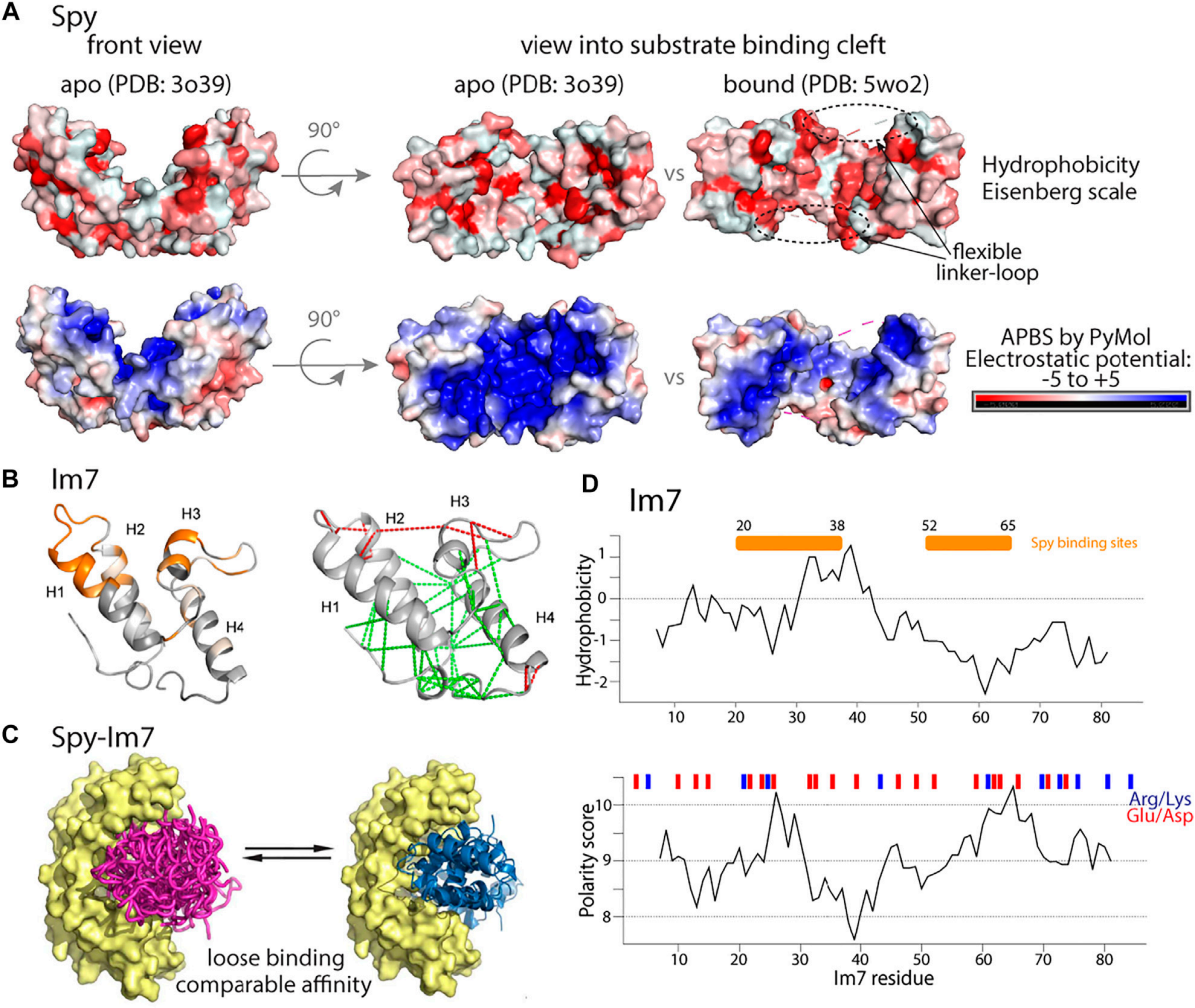
Properties of periplasmic chaperone Spy and its substrate Im7. **(A)** Hydrophobicity **(upper)** and electrostatic potential **(lower)** of the Spy chaperone in its apo or peptide-bound conformation show distinct patterns. **(B)** Chemical shift perturbations mapped on the structure of Im7 (PDB: 1AYI) upon adding Spy chaperone He et al. (2016) are shown in the left panel. In the right panel, structurally frustrated contacts (red lines) mapped on the Im7 structure. Figure adapted from He et al. (2016). **(C)** Representation of chaperone Spy (in yellow) bound to ensemble state of unfolded Im7 (purple) or to folded Im7 (blue). Figure adapted from He et al. (2016). **(D)** Upper: Hydrophobicity prediction of the full length Im7 client protein (Kyte-Doolittle scale). Lower: Polarity prediction (Grantham scale). Red and blue bars denote position of negatively and positively charged residues. The Spy binding site He et al. (2016) is shown with an orange bar. Figure 5B,C has been adapted from He et al. (2016) with permission from Science Advances.

When Spy binds client proteins (as tested with Im7), it does not undergo large structural alterations. The main change is an increased flexibility of the linker loops; this increased loop flexibility may facilitate the interaction with different substrate conformations Salmon et al. (2016). Remarkably, it was found that a more flexible mutant of Spy, with mutations in client-binding cradle shown up to seven-fold higher chaperone activity Quan et al. (2014), suggesting that increased flexibility is important for tight client binding. Furthermore, the polar and charged surface of Spy’s binding cradle is changed (Figure 5A), forcing the conformational changes of the client while the client is still bound Horowitz et al. (2016).

In the complex, the client interacts primarily through a part that forms one face of the folded state (residues ca. 20–38 and 52–65), and the remainder of Im7 adopts essentially a native-like conformation in the complex He et al. (2016), as judged by NMR chemical-shift perturbations (CSPs) (Figure 5B left panel). The client protein was found to be destablized, i.e., more dynamics, as evidenced by hydrogen/deuterium exchange. Spy spatially compacts the conformational ensemble of its substrates. The binding is entropy (rather than enthalpy) driven, presumably due to the release of water molecules that are ordered in the apo state He et al. (2016).

He et al. have also prepared a triple mutant of Im7 which is unable to fold, and observed how this unfolded client interacts with Spy He et al. (2016). The mutant binds to Spy with K_d_ = 0.3 µM affinity (the wt Im7 has a somewhat higher K_d_ = 2 μM; Figure 5C). The Spy-bound Im7 mutant shows the hallmark features of a random-coil polypeptide, and the interacting part of this Im7 includes the residues that are most involved in complex formation in the wild-type Im7, but also residues beyond this stretch, i.e., the binding sequence on the unfolded Im7 mutant is less well defined than on the wild-type Im7.

What drives the interaction between Im7 and its chaperone? When inspecting the biophysical properties of the residues of Im7 that are involved most in the interaction (20–38; 52–65) no clear relationship with the hydrophobic character or the presence of charges appears (Figure 5D). The interaction between the chaperone and the clients involves both hydrophilic and hydrophobic residues. Interestingly, the sites of Im7 that interact most strongly with Spy (residues ca. 20–38 and 52–65) correspond well to the sites for which the local frustration is highest in the native state (Figure 5B). Similar observations have been made with a different client, SH3, bound to Spy He and Hiller (2019). Thus, simple sequence properties do not seem to be the driving force for interactions, and it is rather the properties of the folded state, in particular the local frustration that plays an important role for the interaction of wild-type Im7 and its chaperone. Of note, the client protein in this case, wild-type Im7, appears to be already close to its native fold when it interacts with Spy. For the Im7 mutant that is unable to fold, the argument on structurally frustrated sites does not hold; in this case, a combination of hydrophobic and charged residues appears to be required for binding.

Do these client proteins behave differently when bound to different chaperones? This question was investigated by binding Im7 or SH3 to Skp or SurA He and Hiller (2018). Interestingly, a very similar pattern of CSPs is observed, both for the predominantly folded wild-type Im7 and the unfolded triple mutant, as well as for SH3, showing that the interaction with chaperones is rather independent of the exact details of the chaperone. One may argue that these three chaperones are similar to some extent, an amphipathic binding surface of both hydrophobic and polar residues. Further investigations with chaperones of more divergent properties (e.g., a highly hydrophobic surfaces) would allow clarifying how binding depends on polar/charge/hydrophobic properties.

### 5.3 Hsp90 Interaction With an Intrinsically Disordered Client

The ATP-dependent 90 kDa heat-shock protein family (Hsp90) has been extensively studied [see e.g., reviews by Taipale et al. (2010), Biebl and Buchner (2019)], and represents in itself a field that is far too vast to grasp in this review. The Hsp90 chaperones act in the late stage of protein folding (after Hsp70) and take care of a diverse substrate pool, including intrinsically disordered proteins (IDPs). We focus here only on one insightful complex of cytosolic Hsp90 with the IDP Tau, which has been investigated by NMR and small-angle X-ray scattering (SAXS) methods Karagöz et al. (2014) and EPR methods Weickert et al. (2020). Even though this review focuses on ATP-independent chaperones, we find it insightful to discuss this particular complex, formed by Hsp90 and Tau in the absence of ATP, where the ATP-independent holdase function is of main interest. Hsp90 aids Tau’s association with microtubules or its degradation, and in that way plays a protective role against Tau’s aggregation which is present in certain neurodegenerative diseases Medeiros et al. (2011).

Tau binds Hsp90 with a dissociation constant in the low micromolar range through its microtubule-binding part, including the aggregation-prone repeat region Karagöz et al. (2014). Although the authors have not determined the life time explicitly, the Tau-concentration-dependent NMR and fluorescence signal shows a behavior characteristic of the so-called fast exchange regime. This observation points to on/off rate constants in the µs-ms regime, i.e., the Hsp90-Tau complex forms transiently (as opposed to e.g. the complexes of small TIM chaperones with membrane-protein precursors, section 5.1).

**FIGURE 6:**
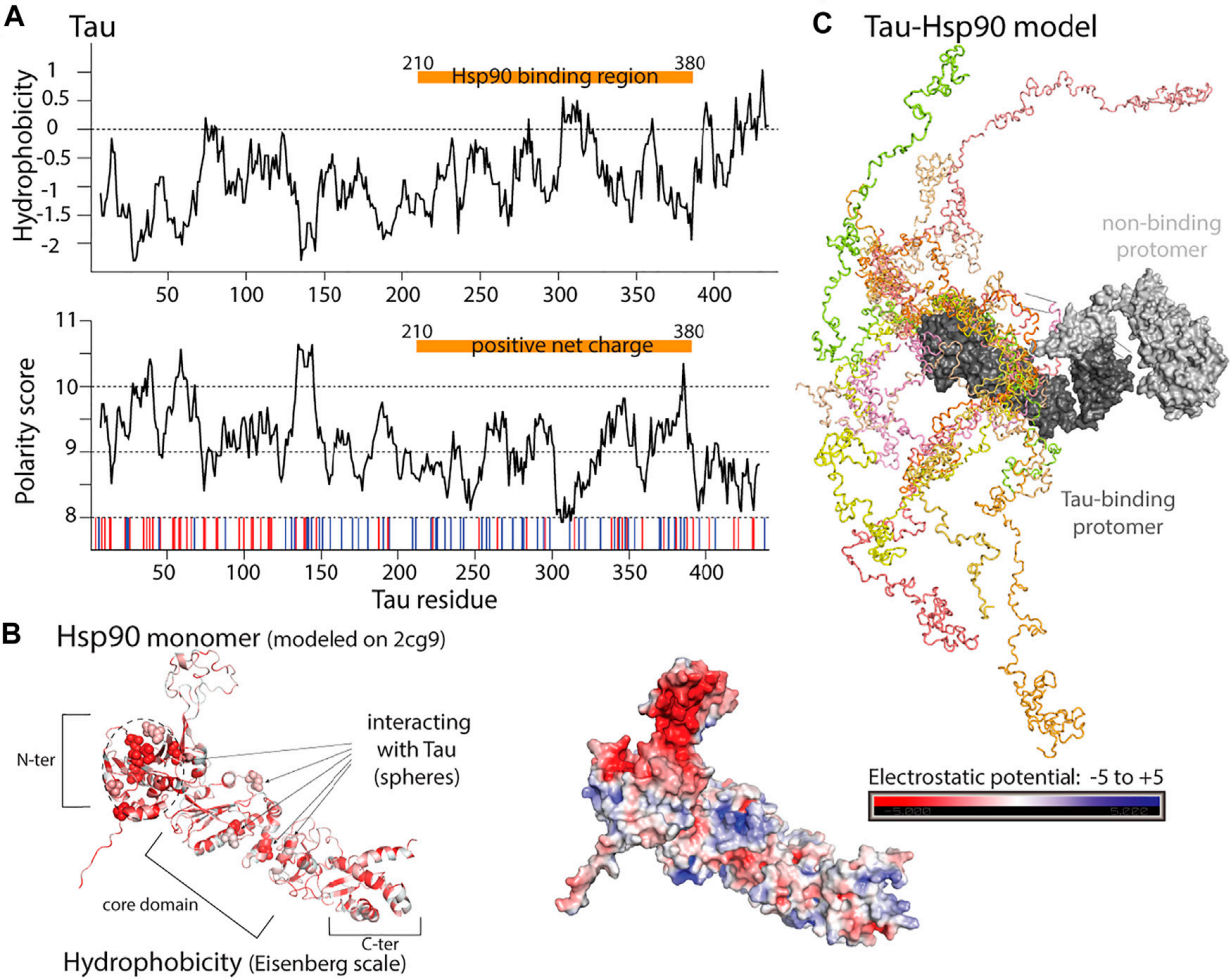
Properties of human Hsp90 and one of its clients, the intrinsically disordered protein Tau. **(A)** Upper: Hydrophobicity prediction of the full length Tau (isoform F) client (Kyte-Doolittle scale). Lower: Polarity prediction (Grantham scale). Red and blue bars: negatively and positively charged residues. Orange bar: Hsp90 binding region. **(B)** Hsp90 has hydrophobic residues distributed over an extended Tau binding surface, shown in red based on Eisenberg hydrophobicity scale in the model on the left, and negatively charged surface centered near the Tau binding region **(right).** Residues shown to be involved in Tau binding [**left,** Karagöz et al. (2014)], represented as spheres. Model of human Hsp90, built with the Swiss-Model server Waterhouse et al. (2018) using the PDB structure of yeast Hsp90 bound to Sba1 (PDB:2cg9) as a template. **(C)** Representation of different Tau conformations (in different colors) bound to Hsp90 chaperone (dark gray), from NMR and SAXS data. The second Hsp90 protomer, not bound to Tau is shown in light gray. Model provided by Dr. Karagöz Karagöz et al. (2014).

The parts of Tau that appear to be the most important motifs (as seen from NMR data) contain hydrophobic residues (amino acid types Leu, Ile, Val, Phe and Tyr), and these sequences have a positive net charge, comprising several Arg and Lys residues (Figure 6A). This part of Tau binds to an extended surface of the chaperone (Figure 6B). The interaction surface on the chaperone has mixed properties: it comprises hydrophobic residues, but they are scattered, rather than forming a continuous hydrophobic patch; the authors claim that this scattered nature may ensure that it can make a large number of low-affinity contacts, and that it may also prevent Hsp90 from self-aggregation. The domains that are involved in the binding (N-ter and middle domains) have an overall negative charge, which shall complement the positive net charge of its client, but the binding site itself has a mixed positive/negative potential. Thus, it appears that the Hsp90/Tau interaction is based on a mixture of rather scattered hydrophobic and charge interactions. Hsp90-bound Tau is in an extended, unfolded and dynamic ensemble (Figure 6C). Electron paramagnetic resonance data show that Tau in isolation is also very dynamic, but has a tendency to fold back on itself (“paper-clip like”); when bound to Hsp90, Tau is extended further than in isolation, and the Hsp90-Tau interaction exposes Tau to oligomerization involving the two C-terminal pseudorepeats Weickert et al. (2020).

### 5.4 The Cytosolic Chaperone SecB

In Gram-negative bacteria, secretory proteins are synthesized on the cytoplasmic ribosome and targeted for post-translational translocation through the inner membrane. Nascent polypeptide chains of these proteins are recognized and transported to the Sec machinery by certain cytosolic chaperones [reviews on Sec machinery by Lycklama a Nijeholt and Driessen (2012), Chatzi et al. (2013), Tsirigotaki et al. (2017)]. The current model is that the ATP-independent SecB chaperone carrying an unfolded client protein binds to SecA in the cytosol and that this ternary complex interacts with the membrane-bound Sec machinery, after which the SecB dissociates, i.e. the client protein is handed over from SecB to the Sec complex [Hartl et al. (1990), Suo et al. (2015)]. [SecB also has other roles reviewed by Sala et al. (2014)].

An early model of client-protein recognition by SecB chaperone based on peptide-binding assays and stopped-flow fluorimetric experiments suggests that the primary interaction is of ionic nature; initial binding could cause a conformational change in SecB, exposing its hydrophobic areas which would then further strengthen the interactions with the substrate protein (Randall, 1992; Stenberg and Fersht, 1997). It was shown that SecB exhibits a preference for unstructured stretches of polypeptides, that contain both basic and aromatic residues (Rüdiger et al., 1997; Knoblauch et al., 1999) and that it binds its clients in a 1:1 ratio [1 SecB tetramer per 1 unfolded client; Lecker et al. (1989), Hardy and Randall (1991), Stenberg and Fersht (1997)] with a 30 µM affinity [obtained by calorimetric titration of SecB into a solution of maltose-binding protein (MBP) at 7°C Randall et al. (1998)]. Huang et al. (2016) reported affinities for binding of unfolded MBP and phosphotase A (PhoA), which are of the order of 0.05 µM (MBP) to 0.5 µM (PhoA); for shorter fragments of the clients (of the order of 30–80 residues) the K_d_ are in the 1–70 µM range.

SecB forms homo-tetramers (dimer of dimers) of ∼68 kDa [PDB: 1qyn, Dekker et al. (2003)]. Huang et al. (2016) have used two different client proteins, the periplasmic maltose binding protein (MBP, 396 amino acids) and alkaline phosphatase A (PhoA, 471 amino acids) as client proteins, and investigated the formed complexes by NMR spectroscopy. MBP and PhoA were chemically unfolded (urea), and while they fold upon removal of urea in the absence of chaperone, SecB keeps them in an unfolded state.

NMR spectroscopy experiments showed that both MBP and PhoA remain in an unfolded conformation upon binding SecB Huang et al. (2016): NMR spectra of the SecB-bound clients strongly resemble those of the urea-denatured unfolded state. The authors performed a structure calculation of the complex based on distance measurements between the chaperone and client [nuclear Overhauser effect (NOE) and paramagnetic relaxation enhancement data]. Interestingly, the authors performed a structure calculation akin to what one generally does when determining the structure of a folded protein, i.e. attempting to determine a single state. Conceptually, their approach of structure determination implies that the energy landscape has a well-defined minimum (cf. Figure 2), which contrasts e.g. the cases of TIM and Skp, where an explicit ensemble is the only way to realistically represent the complex. Whether this implicit single-structure assumption is justified is not within the scope of this review. Based on the resulting structural models, the authors draw the following conclusion on the complex. Distinct parts of the client proteins bind to grooves on the four subunits as well as to a secondary binding site. A slight structural rearrangement of SecB is reported, involving rotation of a helix, which increases the hydrophobic surface; rearrangement of side chains of SecB increases the space available to bulky hydrophobic side chains of the client. The interaction surface is maximized by the client protein being wrapped around the chaperone; the authors state that the simultaneous binding of the multiple sites enhances affinity, but that the binding synergy is not strong.

The interacting regions on the clients are enriched in hydrophobic and aromatic residues; while hydrophobic contacts appear to be the driver of the interaction, several hydrogen bonds line the hydrophobic groove, according to the structure that was determined. Simultaneous substitution of three hydrophobic amino-acids of the SecB binding site were shown to be sufficient for defective binding of the MBP Huang et al. (2016). This observation is remarkably similar to the disruption of client-protein binding of TIM9.10 which can be caused by a single mutation in each Tim subunit Weinhäupl et al. (2018).

In conclusion, the SecB chaperone appears to employ primarily hydrophobic contacts for client binding. As an interesting difference to the other examples, distinct NOE signals are detected and interpreted in the framework of a well-defined binding pose for each of the binding fragments.

### 5.5 The Cytosolic Chaperone Trigger Factor

Trigger factor (TF), an ubiquitous chaperone that forms dimers in the cytosol and binds to the ribosome as a monomer, commonly functions in facilitating co-translational folding of cytoplasmic proteins or in handing them over to downstream foldases for post-translational folding. The structure and function of TF are reviewed, e.g., by Hoffmann et al. (2010). For the purposes of this review we focus on the holdase function of TF and in particular on one complex, the one with model client proteins, PhoA, which was also discussed as a client in SecB studies, and the aggregation-prone G32D/I33P variant of MBP Saio et al. (2014). The complex formation with PhoA was achieved under reducing conditions where PhoA is unfolded, as PhoA requires oxidizing conditions to fold. The affinity is in the low µM range (K_d_), whereby increasing the length of client (full-length PhoA or fragments) leads to enhanced affinity, which points to some degree of binding synergy (avidity). Due to its large size (471 amino acids), PhoA recruits 3 TF monomers Saio et al. (2014). Similar characteristics as for SecB emerged from NMR experiments on TF-bound PhoA and complexes of TF with PhoA fragments: PhoA shows the hallmark features of an unfolded protein when bound to TF, in particular NMR spectra and NMR relaxation parameters characteristic of disordered proteins. It uses only ca. one third of its sequence to bind. A combination of NMR experiments with PhoA fragments allowed structure calculation, which was again performed with methods that aim for a single structure, as discussed in the SecB section above.

The important interactions identified in the resulting complex structures are predominantly hydrophobic in nature, with aromatic residues being mostly involved. A single amino-acid substitution at the hydrophobic substrate-binding sites in TF resulted in a significant decrease in the affinity for PhoA Saio et al. (2014), similarly to TIM9.10 or SecB.

### 5.6 J-Domain Proteins (Hsp40) and Client Specificity

J-domain proteins (JDP) are a component of the important Hsp70-Hsp40/NEF (nucleotide-exchange factor) system. They are often referred to as 40 kDa heat-shock proteins (Hsp40), arguably a misleading name as most JDPs have a mass rather different from 40 kDa. In this tripartite chaperoning system, Hsp70 is the ATP-driven holdase/foldase which binds to a very broad range of substrates. Hsp70 is very promiscuous: the binding sequence has a hydrophobic core (4–5 residues) flanked by basic residues. In the *E. coli* proteome, Hsp70-binding motifs occur statistically every 36 residues Rüdiger et al. (1997). A number of crystal structures of Hsp70 in complex with peptides have revealed a well-defined binding pocket [Mayer and Gierasch (2019), Mayer (2021)]. NEF assists the release of ADP from Hsp70 after ATP hydrolosis Kampinga and Craig (2010). The JDPs have several roles: 1) they stimulate the ATP-hydrolysis of Hsp70, which in turn leads to tighter binding of Hsp70 to its client, and 2) they bind to clients and hand them to Hsp70. The latter generally has higher client affinity; for example, a fragment of a mitochondrial outer-membrane protein binds to yeast Hsp70 about ten-fold stronger than it does to the JDP Ydj1, suggesting the directional transport, from JDP to Hsp70 Jores et al. (2018).

The ability of a given Hsp70 (highly promiscuous) to interact with different JDP co-chaperones brings the outstanding versatility to the Hsp70 system, engaged in a myriad of cellular processes [Kampinga and Craig (2010), Craig and Marszalek (2017), Barriot et al. (2020), Mayer (2021)]. Consequently, most species have many more JDP genes than Hsp70 genes; e.g., the cytoplasm of *Saccharomyces cerevisiae* contains 13 different JDPs (Apj1, Xdj1, Ydj1, Caj1, Djp1, Hlj1, Sis1, Cwc23, Jjj1, Jjj2, Jjj3, Swa2, and Zuo1) but only two classes of Hsp70s, Ssa (SSA1–4) and Ssb (SSB1–2) (Sahi and Craig, 2007). JDPs by themselves can suppress protein aggregation, and in some cases they function in the cell without the involvement of Hsp70 [Kampinga and Craig (2010), Craig and Marszalek (2017)].

**FIGURE 7:**
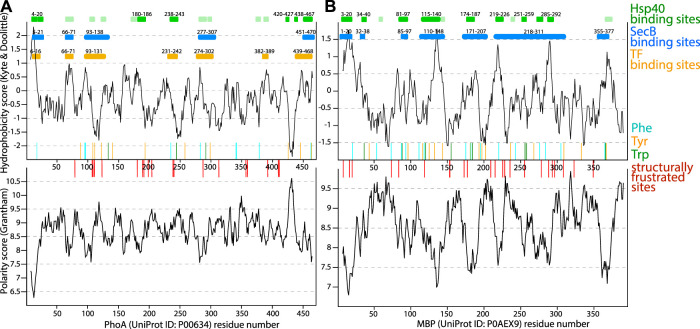
Properties of the model client proteins alkaline phosphatase A (PhoA) and maltose-binding protein (MBP). **(A)** Plot of hydrophobicity **(upper panel)** and polarity **(lower panel)** of PhoA as a function of its residue number. Same as for the plots shown previously, plots were obtained with Expasy ProtScale bioinformatic tool (window size 13). **(B)** Hydrophobicity **(upper)** and polarity **(lower)** plots of MBP as a fuction of its residue number. In orange colored bars, experimentally determined binding site of TF chaperone on PhoA client protein are shown Saio et al. (2014). Binding sites of SecB chaperone Huang et al. (2016) and of Hsp40 chaperone Jiang et al. (2019) on PhoA and MBP are shown with blue and green colored bars, respectively. For each client protein, position of aromatic residues (Phe in cyan, Tyr in orange and Trp in green) are indicated on the plots, along with the position of structurally frustrated residues (in red). Measuring of how favorable a particular amino-acid contact is in the protein structure was calculated with Frustratometer Server Ferreiro et al. (2014) where contacts with frustration index <−1 were classified as highly frustrated and indicated in this figure.

Common to all JDPs is the presence of a J-domain, a ca. 80-residue helix-bundle domain; besides this defining common feature, there is a wide variety of additional domains. The most ubiquitous classes of JDPs (type I and II) comprise β-barrel C-terminal domains I and II (CTD-I, CTD-II) and a dimerization domain at the extreme C-terminus; thus, these JDPs act as dimers, which increases the number of interaction sites to clients (as compared to monomers). Type I JDPs carry an additional zinc-finger domain Kampinga and Craig (2010). The JDPs that do not fulfill these definitions are called type III, with a large variety of domains. Some JDPs, especially of type III, are highly client specific, and bind only a single protein [Fotin et al. (2004), Vickery and Cupp-Vickery (2007)]. To do so, however, they use a dedicated domain for recognition; however, most type I and type II JDPs have a larger clientome. Nonetheless, specific involvement of certain JDP chaperones in binding different clients is common. For example, How do JDPs recognize their clients? Interesting insight comes from an NMR study of several JDPs (type I and type II) with the model client proteins PhoA and MBP, which were introduced in the SecB and TF sections above Jiang et al. (2019). Akin to their behavior on SecB and TF, the two client proteins bind the JDP chaperones in a largely unfolded dynamic conformation devoid of secondary structure. Making use of the changes observed for NMR signals of the clients, the authors identified regions of PhoA and MBP which bind to the JDP. These are enriched in hydrophobic residues and comprise at least one aromatic residue; a negatively charged residue preceding the aromatic residue increases the affinity. These distinct stretches are shown in Figures 7A,B. The native-state secondary structure of these interacting stretches varies, suggesting that the final secondary structure is unimportant for binding.

The binding sites on the JDPs are located to the C-terminal domains I and II, within hydrophobic grooves in these domains; the sequence preferences of these two grooves slightly differ. However, not all the tested JDPs use both CTDs, and the authors propose how client specificity in the JDP family may arise, namely through the fact that not all JDPs use both CTDs, and the different preferences of the two CTDs within a JDP.

Structure calculation in that study involved a complex protocol based on calculations with small fragments; it is challenged by the inherent dynamics of the complex and the difficulty to identify with certainty which part of the client binds to which part of the JDP (Jiang et al., 2019). From NMR relaxation-dispersion (RD) data and bio-layer interferometry (BLI) data the authors state that the life time of the complexe is in the millisecond range. To us, this estimation is not entirely clear, as their BLI data rather point to slower on/off kinetics [seconds; Figure S10 of Jiang et al. (2019)], and the RD data were obtained only for a small fragment of PhoA, not representative of the entire client.

Other studies reported that when a given JDP engages with different client proteins, it may use different parts to do so. For example, both DNAJB6 and DNAJB8 bind stretches of glutamine (polyQ) in an amyloid-forming polypeptide (GAMKSFQ_45_F) that is largely disordered with a tendency to form β-turns and aggregate. For the interaction with these polyQ stretches, a Ser/Thr-rich region of the JDP is essential Kakkar et al. (2016b). The same JDPs also bind to another target, a mutant of the E3 ubiquitin ligase Parkin, a protein comprising globular folded ubiquitin-like and RING domains in its native state. Interestingly, for the interaction with this Parkin mutant, the Ser/Thr-rich region of the JDP is dispensable Kakkar et al. (2016a). Thus, this JDP has different modes of client interaction. Of note, the two described clients are structurally different, one is intrinsically disordered and the other one folds to a well-defined 3D structure, which may be responsible for recruiting different parts of the JDP.

## 6 Conclusion: Emerging Patterns of Client-Chaperone Interactions

The function of chaperone proteins and the mechanisms by which they recognize, bind and fold their client proteins has been of great interest to structural biologists for more than 3 decades [Ellis (1990), Neupert et al. (1990), Hartl et al. (1992), Kelley and Georgopoulos (1992), Jakob and Buchner (1994), Horovitz (1998), Feldman and Frydman (2000)]. Crystallography has been crucial to obtain structures of the apo states but it has so far turned out to be very limited when it comes to characterizing structures of chaperones with full-length client proteins, due to the heterogeneity and dynamics. Only recently, and by combined efforts from several techniques (NMR, SAXS, SANS, FRET, MD, often integrated), we are getting atomic-level insights into the interactions that underlie chaperone–client interaction.

It is often assumed that the mechanisms by which chaperones hold polypeptides is by binding to hydrophobic stretches, thereby protecting them from self-aggregation in the aqueous environment. The selection of chaperone complexes that we have reviewed here shows that chaperone–client interaction is more complex than being limited to hydrophobic interactions, and we summarize here the emerging view.

The kind of interactions that is important for the complex formation depends on the nature of the client protein and the degree at which it is folded when encountering the chaperone. Among the clients we have encountered here were proteins which are unable to fold (in the environment where they bind the chaperone), while others can fold, or are even already close to the folded state. Along this continuum from disordered to folded proteins the way how chaperones and client proteins interact with each other necessarily differs. For proteins that are close to their native state already, the interaction sites are essentially those sites that are least stable. Structurally frustrated sites generally correspond to these least stable parts of proteins (Ferreiro et al., 2014), therefore there is a good correspondence between the sites with highest structural frustration and the chaperone-binding sites. This was found for Im7 and SH3, two proteins which are known to populate partially unfolded intermediate folding states in solution, and which are close to their native state when bound to Spy (see section 5.2.5). The exact nature of the amino acids or the structural motif does not appear to be the determining criterion in such cases; in the Im7 and SH3 cases it is a mix of charged, polar and hydrophobic residues which binds the chaperone.

On the opposite extreme of this spectrum are proteins which are unable to fold, and which comprise parts that need to be shielded from the solvent to prevent aggregation. The mitochondrial membrane proteins are such a case (section 5.1), which bind to hydrophobic groove of the chaperone with their hydrophobic transmembrane parts, retaining only a small helical tendency in an otherwise elongated conformation. Reducing the hydrophobic nature of the chaperone by a single hydrophobic-to-charged mutation can totally abrogate the ability to hold the client. However, even for such unfolded clients, not only hydrophobic contacts are important, as highlighted by the cases of the complex formed by Hsp90 and the intrisically disordered (and aggregation-prone) Tau (section 5.3), or the SurA-Omp and Skp-Omp cases (section 5.2.1 and section 5.2.2); these chaperones present a binding surface that comprises many charged residues, and electrostatic interactions contribute to the complex formation. In the case of the (unfolded) clients PhoA and MBP binding to SecB, TF and JDPs, the frustrated sites do not appear to be particularly involved in binding (Figure 7); arguably, this can be expected, as the clients are totally unfolded, and thus their primary structure is important, but not the structure of the folded state.

The different types of interactions (hydrophobic, polar, electrostatic) may also contribute at different points along the complex-formation process: for the Skp chaperone, for example, initial binding is most likely driven by electrostatic interactions and the client is then encapsulated additionally by the hydrophobic interactions [Qu et al. (2007), Qu et al. (2009)]. Similarly, rapid initial binding of the Im7 substrate by the Spy chaperone is thought to be achieved thought the electrostatic (not hydrophobic) interactions, and the complex is further strengthened by the hydrophobic interactions in the binding site Koldewey et al. (2016). Hereby, locally frustrated, and thus inherently unstable/unfolded parts of the partly folded client are particular hotspots of interaction with the chaperone He and Hiller (2019). The self-folding of the client on the chaperone surface triggers client release, and thereby the chaperone-client interaction is terminated without the need for any particular trigger event Koldewey et al. (2016).

ATP-driven chaperone machineries exploit this combination of interaction types in a more active manner: Hsp60 chaperonins “capture” their clients through hydrophobic interactions close to the chamber entry, and then, upon ATP hydrolysis and allosteric closure of the chamber including rotation of some helices, the client protein finds itself in a much more hydrophilic environment (Yebenes et al., 2011; Lopez et al., 2015).

Client-specificity of a chaperone, and chaperone-specificity of a given client protein are important in the complex environment of the cell, where the right cellular localization of a protein is also related to the way it is transported to e.g., cellular compartments. The question about specificity comprises two questions: does a given client protein interact in similar ways with different chaperones? And does a given chaperone hold different client proteins in a similar manner? The examples discussed here show that there are clearly similarities when a client binds to different chaperones: for example, OMPs are similarly behaved (namely unfolded) in Skp and SurA; PhoA and MBP engage with similar stretches along their sequence when binding to SecB, TF and Hsp40; the Tim23 client wraps its hydrophobic transmembrane part around the clefts of TIM9.10 and TIM8.13 similarly. However, in detail the interactions differ, which opens the possibility for introducing specificity. We have discussed the case of Tim23-binding to TIM8.13 vs. TIM9.10, where only one of the two (TIM8.13) is able to engage in hydrophilic contacts with a 100-residue long stretch of the client, while the other one is better in binding hydrophobic stretches; accordingly, these two chaperones are able to diversify their clientome Sučec et al. (2020). The Hsp40 (JDP) chaperone system is another important case, and we discussed the example where a given Hsp40 uses a Ser/Thr rich part when interacting with some clients, but not with others (section 5.6). Along these lines, we described the NMR study that suggested that different JDPs use either only one or two of the CTDs to engage with a given client, thus also allowing for specificity.

In the context of specificity, it must be mentioned that the cellular localization is also of central importance. Chaperones are often positioned at strategic points, for example on the ribosome Craig and Marszalek (2017) or near the exit of translocation pores [e.g. in the two mitochondrial membranes Shiota et al. (2015), Craig (2018)]. Interactions of the chaperones with these machineries (ribosome, translocases) keeps them right at the location where they are required.

Furthermore, chaperones collaborate, and a given client protein is often handed over from one chaperone to the next. Such interaction networks can either involve functionally redundant chaperones or a step-wise substrate transfer within chaperone cascades. For example, bacterial OMPs are handled by different periplasmic chaperones. A holistic view on the periplasmic chaperone network was obtained from a mathematical model that integrated available experimental information from *in vivo* and *in vitro* studies. From these simulations, the authors concluded that functional robustness does not necessarily rely on the concept of specific pathways [Costello et al. (2016), Chum et al. (2019)]. On the other hand, within the GET pathway, tail-anchored membrane proteins (TA-MBPs) are transported to the ER membrane via a step-wise substrate transfer from highly promiscuous Hsp70 (Ssa1 in yeast) to the selective Get3 that traps TA-MBPs for membrane insertion. Such a cascade, engaging more specialized chaperones with increasing affinity allows for efficient, selective, and unidirectional targeting of nascent TAs, while protecting them from reaction with other cytoplasmic chaperones [reviewed in Shan (2019)]. In a similar way, a network of chaperones including different Hsp40s, Sti, Hsp70 and Hsp90 is important for safeguarding mitochondrial precursor proteins across the cytoplasm Bykov et al. (2020).

A common theme found in many chaperone systems is their oligomeric nature, found for example in small TIMs (hexamer), Skp (trimer), Hsp40 (dimer), Spy (dimer) or Hsp60 (14- or 16-mer). The dimerization represents several advantages for a chaperone. Most importantly, it enables the chaperone to present a larger binding surface which is often also used as a cavity-like architecture. Through the multiple interaction sites with the client, the oligomeric chaperone strongly enhances its affinity to the client by avidity. When the subunits are allosterically coupled to each other, such an oligomeric machinery may perform even concerted large scale movements; positive intra-ring allostery and negative inter-ring allostery in chaperonins are an example of the functional complexity that can be achieved from relatively small building units Yebenes et al. (2011). The oligomeric nature often implies that subunits can go in an out in a dynamic manner. In small TIM chaperones Weinhäupl et al. (2021) and Skp Mas et al. (2020), the oligomeric chaperones are in equilibrium with monomeric subunits. Together with a protease system that clears exclusively the monomers Baker et al. (2012) and replenishment by newly synthesized subunits, the concentration of chaperones can thus be adjusted very efficiently. This may present a simple way to adjust the chaperone level to the state of the cell. The dynamic monomer-oligomer equilibrium also presents a direct way of regulating the chaperone activity. In the Skp system, the presence of client proteins shifts the equilibrium from the monomeric to the oligomeric state; thus, the presence of clients generates a higher effective chaperone concentration, and therefore the chaperone activity is very rapidly adjusted to the needs. Interestingly, in some chaperone systems, the inverse process occurs: the chaperone exists in a high-oligomer “resting state”, and the presence of client leads to a deoligomerization of the chaperone into smaller subunits and activation of its chaperone activity [Haslbeck et al., 1999; Jehle et al., 2010].

A central property of all the chaperones discussed here is their dynamic nature. These complexes are often held together by a multitude of individually weak interactions, which, due to their large number, can result in a strong overall affinity. Dynamics is important in chaperone-client complexes for several reasons. First, the multi-conformation dynamical ensemble results in a more favorable entropic contribution for binding than a single conformation would do. In contrast to complexes of folded proteins, which are rich in highly specific interactions (such as salt bridges) which make a large enthalpic contribution to binding, chaperone-client complexes often do not have such interactions. Thus, while the free energy of binding in rather rigid complexes of folded proteins is dominated by a strong enthalpic component (and an entropic penalty), this is not the case for chaperone-client complexes, and the entropic component shall be favorable (or at least less disfavored) than for complexes of folded proteins. Second, as discussed above, dynamics in chaperone complexes can lead to high overall affinity (particularly important for holdases of highly aggregation-prone client proteins), while avoiding high energy barriers for release (important for efficient release).

The details of the dynamics of a given client bound to different chaperones, or of different clients bound to a given chaperone certainly differ. Such differences may provide a further layer by which the cell can differentiate client proteins. Technically, different dynamic behaviors of diverse clients means that their experimental characterization has to be adapted. For example, the NMR signature of a protein sliding in a chaperone’s binding pocket depends on the time scale of this motion; if it falls into the millisecond range, NMR line broadening is induced, which challenges the extraction of information about the conformation and inter-molecular contacts. Due to the broad range of dynamics in these complexes, their characterization generally requires multiple techniques, and it will certainly continue to be a playground of integrated structural biology, where advanced computational methods, such as explicit ensembles derived from MD simulations are confronted with a multitude of experimental observables.

It must be stressed that we are only starting to decipher the chaperone function at the atomic level, and that it can be expected that there is much more diversity than what appears from the examples described here. The proteins that have been selected in these studies may well provide a biased view: the clients are often model proteins, or artificially denatured by urea or mutations. The complexes have also rather divergent life times and affinities: Skp or TIM chaperones capture their clients for many hours, while Spy–Im7 or Hsp90–Tau complexes have life times in the millisecond range. Whether this impressive factor of 10^5^–10^6^ difference is related to the fact that the former rely much more on hydrophobic interactions, or whether the difference comes from the interaction surface area or the architecture of the chaperone is not clear and needs further investigation.

Although we have just scratched the surface of the molecular and structural features of these complexes, the features that these examples have revealed will provide an important foundation as the community will explore more complex targets. Moving towards such more complicated and larger clients, and to higher-order complexes is certainly on the “to do list” for the field. It is becoming increasingly clear, for example, that many JDP clients are mature folded proteins, and that the JDPs remodel large multiprotein complexes, acting on the protein-protein interactions within these complexes. Studying such complexes will likely shed light onto new mechanisms. Another field of central importance is the one of membrane-insertion machineries. The hydrophobic membrane environment corresponds to physico-chemical properties very different from the aqueous solution in which most chaperones are studied currently. Continued technical improvements will be important for tackling these complex membrane-integrated/membrane-associated machineries.
